# A WRKY Protein, MfWRKY40, of Resurrection Plant *Myrothamnus flabellifolia* Plays a Positive Role in Regulating Tolerance to Drought and Salinity Stresses of *Arabidopsis*

**DOI:** 10.3390/ijms23158145

**Published:** 2022-07-24

**Authors:** Zhuo Huang, Jiatong Wang, Yuan Li, Li Song, Duo’er Chen, Ling Liu, Cai-Zhong Jiang

**Affiliations:** 1 College of Landscape Architecture, Sichuan Agricultural University, Chengdu 611130, China; wangjiatong@stu.sicau.edu.cn (J.W.); yuanlisci@outlook.com (Y.L.); imsongli@stu.sicau.edu.cn (L.S.); 2020210028@stu.sicau.edu.cn (D.C.); myliuling1994@163.com (L.L.); 2 Department of Plant Sciences, University of California Davis, Davis, CA 95616, USA; caizhong.jiang@usda.gov; 3 Crops Pathology and Genetics Research Unit, United States Department of Agriculture, Agricultural Research Service, Davis, CA 95616, USA

**Keywords:** *Myrothamnus flabellifolia*, drought tolerance, salinity tolerance, WRKY, zinc finger

## Abstract

WRKY transcription factors (TFs), one of the largest transcription factor families in plants, play an important role in abiotic stress responses. The resurrection plant, *Myrothamnus flabellifolia*, has a strong tolerance to dehydration, but only a few WRKY proteins related to abiotic stress response have been identified and functionally characterized in *M. flabellifolia*. In this study, we identified an early dehydration-induced gene, *MfWRKY40*, of *M. flabellifolia*. The deduced MfWRKY40 protein has a conserved WRKY motif but lacks a typical zinc finger motif in the WRKY domain and is localized in the nucleus. To investigate its potential roles in abiotic stresses, we overexpressed *MfWRKY40* in *Arabidopsis* and found that transgenic lines exhibited better tolerance to both drought and salt stresses. Further detailed analysis indicated that MfWRKY40 promoted primary root length elongation and reduced water loss rate and stomata aperture (width/length) under stress, which may provide *Arabidopsis* the better water uptake and retention abilities. MfWRKY40 also facilitated osmotic adjustment under drought and salt stresses by accumulating more osmolytes, such as proline, soluble sugar, and soluble protein. Additionally, the antioxidation ability of transgenic lines was also significantly enhanced, represented by higher chlorophyll content, less malondialdehyde and reactive oxygen species accumulations, as well as higher antioxidation enzyme activities. All these results indicated that MfWRKY40 might positively regulate tolerance to drought and salinity stresses. Further investigation on the relationship of the missing zinc finger motif of MfWRKY40 and its regulatory role is necessary to obtain a better understanding of the mechanism underlying the excellent drought tolerance of *M. flabellifolia*.

## 1. Introduction

Throughout the life cycle, plants are subjected to various environmental stresses, such as drought, high salinity and extreme temperatures. Water deficit and salt stress are among the two major forms of abiotic stress that have a serious impact on plant growth and development. To adapt to such unfavorable environmental conditions, plants have evolved complex mechanisms to respond to these abiotic stresses at phenotypic (height, leaf size, root growth, etc.), physiological and biochemical (metabolism, enzymes, hormones, etc.), and molecular (protein expression, gene expression, etc.) levels [1,2,3].

Transcription factors are specialized proteins that bind to DNA-regulatory sequences, usually localized in the 5′-upstream region of the target genes, to activate or suppress gene transcription. Numerous studies indicated that transcription factor families, such as bZIP, bHLH, WRKY, MYB, and AP2/ERF, are involved in the plants’ responses to abiotic stresses [4,5,6,7,8]. WRKY, one of the largest transcription factor families in plants, is important for plant growth, development, and resistance to external stresses, especially drought and salt [9,10,11]. For example, overexpressing *TaWRKY46* in *Arabidopsis* increased drought tolerance in abscisic acid (ABA)-dependent and ABA-independent ways [12]. Transgenic rice plants overexpressing *OsWRKY8* also exhibited improved drought tolerance [13], while cotton *GhWRKY33* can reduce the drought tolerance of transgenic *Arabidopsis* plants [14]. Moreover, overexpression of cotton *GhWRKY34* and *GhWRKY39-1* in *Arabidopsis* and tobacco can significantly improve the salt tolerance [15,16]. Through regulatory functions of the SOS (salt overly sensitive) pathway, overexpression of the *AtWRKY15* and *AtWRKY46* genes in *Arabidopsis* can improve salt tolerance [17,18].

WRKY proteins are unique to plants, with nearly 100 members in *Arabidopsis*. All WRKY proteins are named by the conserved WRKY domain comprised of two prominent structures: a short peptide WRKYGQK (WRKY motif) and the C2H2 or C2HC type zinc finger motif, which is generally highly conserved among family members. In addition, these proteins can specifically bind to the W box motif, which can differentially regulate the expression of various target genes [5]. WRKY proteins can be divided into two categories according to the number of WRKY domains, those with two WRKY domains belonging to class I and most WRKY proteins with only one WRKY domain belonging to class II, both of which have a (C-X_4__–__5_-C-X_22__–__23_-H-X_1_-H) zinc finger structure. There is a class of WRKY proteins containing a zinc finger structure (C-X_7_-C-X_23_-H-X_1_-C) which is different from the first two classes and separately grouped into the class III [5].

WRKY40 together with WRKY18 and WRKY60 have only one WRKY domain and belong to class II WRKY TF. In *Arabidopsis*, they are pathogen-induced and can interact directly with themselves and with each other. They have a partially redundant negative effect on the salicylic acid (SA)-mediated defense but a positive role in the Jasmonic acid (JA)-mediated defense [19]. Additionally, they all respond to ABA and abiotic stress. WRKY18 and WRKY60 could enhance plant sensitivity to ABA, salt, and osmotic stress, whereas WRKY40, on the other hand, antagonizes their effects [20]. Che et al. [21] reported that overexpression of *AtWRKY40* enhanced drought stress responses, presumably by interfering with the reactive oxygen species (ROS)-scavenging pathway and osmolyte accumulation process.

Research has shown that WRKY40 is integral to the growth and development of plants, as well as their resistance to external stresses. Jiang et al. [22] found that *NbWRKY40* might regulate tomato mosaic virus resistance by modulating the expression of SA, leading to the accumulation of callose (a sugar around each sieve pore) in the neck of plasmodesmata, thus interfering with virus movement. Karim et al. [23] determined that *PtrWRKY40* plays a negative role in the resistance of poplar to semi-vegetative fungi; however, it has a positive impact on the resistance of *Arabidopsis* to necrotic vegetative fungi. According to Lin et al. [24]., *PbWRKY40* could play a role in salt tolerance and organic acid accumulation in part by regulating the expression of *PbVHA-B1* Moreover, wheat *TaWRKY40* could positively regulate transcription of *TaGAPC1* to enhance tolerance to drought stress [25]. Studies on kumquat showed that *FcWRKY40* participates in the ABA-signaling pathway and acts as a positive regulator of salt tolerance by regulating genes involved in ion homeostasis and proline biosynthesis [26]. *ZmWRKY40* is induced by drought, high salinity, high temperature, and ABA. Transgenic *Arabidopsis* plants with overexpression of *ZmWRKY40* are more drought tolerant [27]. These results indicated that WRKY40 may positively regulate plant tolerance to abiotic stress.

*Myrothamnus flabellifolia* is the only woody resurrection plant found so far. Various aspects of drought tolerance of *M. flabellifolia* have been extensively studied, including its morphological structure [28,29,30], physiological and biochemical characteristics [31,32,33], as well as its physiological and metabolic pathways [34] during rehydration, but research on its drought tolerance mechanism is limited. Ma et al. [35] examined the transcriptome of *M. flabellifolia* and found that approximatel 295 TFs are responsive to dehydration. *MfWRKY40* was evidently upregulated in the early dehydration stage, suggesting that it may participate in response to a water deficit [35]. In this study, we cloned the *MfWRKY40* and overexpressed it in *Arabidopsis* to investigate its potential roles in tolerance to drought and salt.

## 2. Results

### 2.1. Cloning and Sequence Analysis of MfWRKY40

**Figure 1:**
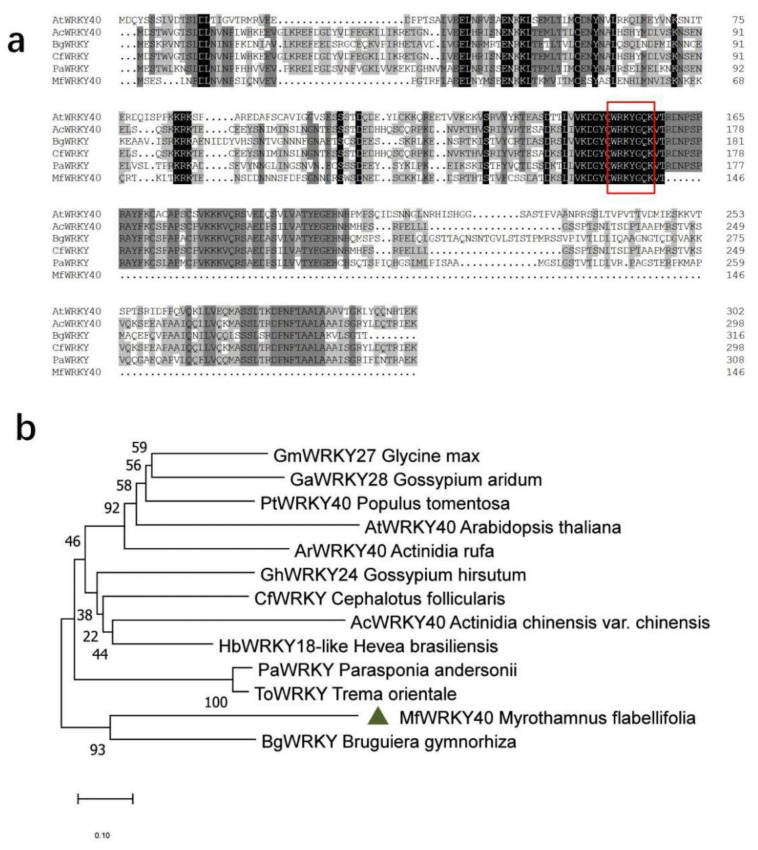
Multiple sequence alignment (**a**) and phylogenetic analysis (**b**) of MfWRKY40 and several highly homologous WRKY proteins. Black and gray shade in (**a**) show identical and similar amino acids, respectively. The WRKY motif was indicated by red box. (**b**) Phylogenetic reconstruction using the neighbor-joining method. The accession numbers for the sequences used were as follows: AtWRKY40 (AT1G80840) of *Arabidopsis thaliana*; ArWRKY40 (GFZ11870.1) of *Actinidia rufa*; PtWRKY40 (AZQ19202.1) of *Populus tomentosa*; GaWRKY28 (AIY62465.1) of *Gossypium aridum*; GmWRKY28 (ABC26917.1) of *Glycine max*; AcWRKY40 (PSS01081.1) of *Actinidia chinensis* var. *chinensis*; HbWRKY18-like (XP_021659791.1) of *Hevea brasiliensis*; GhWRKY24 (AGV75937.1) of *Gossypium hirsutum*; CfWRKY (GAV83654.1) of *Cephalotus follicularis*; PaWRKY (PON74566.1) of *Parasponia andersonii*; ToWRKY (PON99223.1) of *Trema orientale*; and BgWRKY (BAG15874.1) of *Bruguiera gymnorhiza*.

The cDNA sequence of the coding region of *MfWRKY40* (unigene ID: comp48667_c1__seq16) was cloned from *M. flabellifolia* by PCR amplification. The obtained nucleotide sequence is 441 bp in length and possesses an open-reading frame (ORF) encoding 146 amino acids. The calculated isoelectric point of MfWRKY40 is 6.15, and the deduced molecular weight is 16.8 kDa. A blastp search against the *Arabidopsis* genome data indicated that it showed the highest degree of homology to *AtWRKY40* of *Arabidopsis*. Multiple alignment of the deduced amino acids of *MfWRKY40* and several highly homologous WRKY proteins showed that all of them have a conservative “WRKYGQK” domain, indicating that it belongs to WRKY TF (Figure 1a). However, MfWRKY40 lacks the zinc finger structure and is significantly shorter than other WRKYs. Phylogenetic analysis indicated that MfWRKY40 was most homologous to a WRKY protein of *Bruguiera gymnorhiza* (Figure 1b).

### 2.2. MfWRKY40 Is Localized in the Nucleus

**Figure 2:**
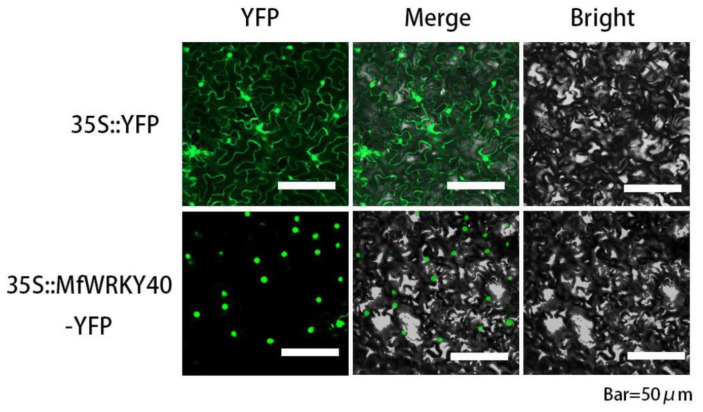
Subcellular localization of MfWRKY40. YFP, yellow fluorescent protein.

We transiently expressed 35S::MfWRKY40-YFP in tobacco leaf epidermal cells. Confocal microscope observation showed that fluorescence of 35S::YFP could be detected in the whole cell, while strong fluorescence appeared specifically in the nucleus of 35S::MfWRKY40-YFP transformed cells (Figure 2). These results proved that MfWRKY40 is located in the nucleus, although no sequence for nuclear localization signal is predicted.

### 2.3. Overexpressing MfWRKY40 Increased Drought and Salt Tolerance

To analyze the potential role under abiotic stress, we introduced *MfWRKY40* into *Arabidopsis* by *Agrobacterium tumefaciens* mediated genetic transformation driven by the CaMV 35S promoter. T_1_ transgenic *Arabidopsis* lines expressing *MfWRKY40* gene were acquired from kanamycin-resistance screening, and two homozygous positive T_3_ transgenic lines identified by PCR were randomly selected for further analysis.

**Figure 3:**
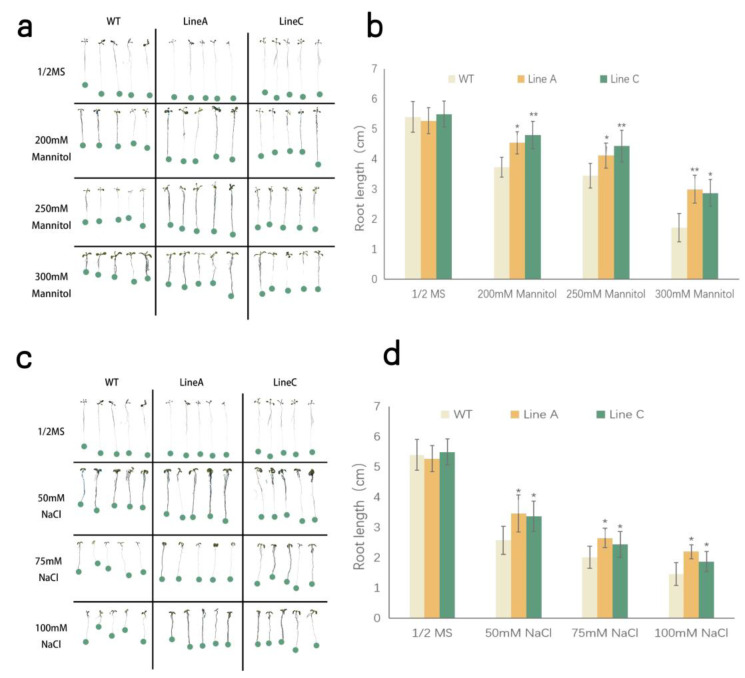
Analysis of drought and salinity tolerance at seedling stage. (**a**,**b**) indicated morphology of transgenic and WT seedlings grown for nine days on 1/2 MS medium with varying concentrations of mannitol and NaCl. (**c**,**d**) indicated root length of corresponding plants under different treatments. Data are presented as mean and SD values (error bar) of three independent experiments. Asterisks indicate significant difference (* *p* < 0.05, ** *p* < 0.01, by independent sample *t*-test) comparing to WT.

To investigate whether *MfWRKY40* is associated with abiotic stress tolerance, WT and transgenic lines were treated by drought and salinity stresses at the seedling and adult stages. For seedling treatment, the seedlings of wild type (WT) and transgenic lines (OE) were planted on solid medium containing different concentrations of mannitol and NaCl. The WT and OE lines showed similar primary root length and lateral root number at normal condition. Under the treatments of mannitol with concentrations from 200 mM to 300 mM, no significant change on lateral root number was found. The root lengths of the WT and OE lines were gradually decreased, but those of line A and line C were 1.22 and 1.29, 1.19 and 1.29, 1.75 and 1.67 folds of those of WT, respectively (Figure 3a,b). Similar to mannitol treatment, two OE lines, A and C, exhibited significantly longer primary roots, which were 1.34 and 1.31, 1.31 and 1.21, and 1.50 and 1.28 folds of those WT under 50 mM, 75 mM, and 100 mM NaCl treatments, respectively (Figure 3c,d).

**Figure 4:**
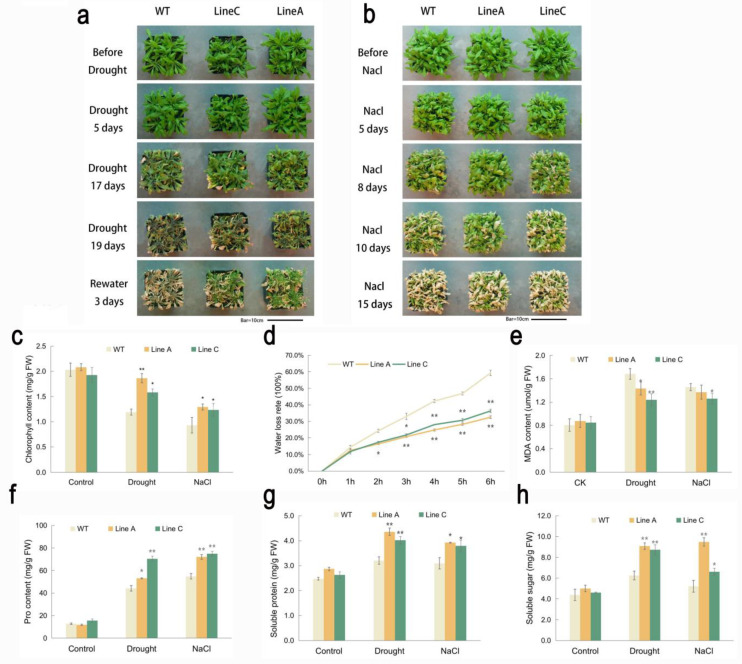
Analysis of drought and salinity tolerance at the adult stage. (**a**,**b**) showed the change of growth status of transgenic and WT plants during the progress of drought and salinity treatments. (**c**–**h**) showed measurements of tolerance-related physiological indexes. Data are presented as mean and SD values of three independent experiments. Asterisks indicated significant difference (* *p* < 0.05, ** *p* < 0.01, by independent sample *t*-test) comparing to WT.

For tolerance evaluation at the adult stage, four-week-old OE lines and WT plants were treated by natural drought (stopping watering) and 300 mM NaCl solution. They were morphologically similar before treatment. At the early stage of drought treatment (five days after stopping watering), the leaves of WT and OE lines began to wither. Seventeen days after stopping watering, the leaf withering became more serious, but the two OE lines were less dehydrated. After 19 days, although almost all plants were severely dehydrated, the two OE lines, especially the line A, were less withered. Three days after rewatering, the WT almost died, whereas the two OE lines were significantly recovered (Figure 4a).

The leaf withering was detected at about day five of the salt treatment. From five to ten days of salt treatment, more withered leaves were found on both the WT and OE lines, but the latter, especially the line A, had more green leaves. At day 15 of the salt treatment, most leaves of the WT and OE lines were severely wilted, but more leaf parts of the line A were still green (Figure 4b). The above results showed that *Arabidopsis* overexpressing *MfWRKY40* exhibited better tolerance to drought and salt stresses.

### 2.4. Measurement of Tolerance-Related Physiological and Biochemical Parameters

Drought and salinity will induce the reduction of the chlorophyll contents in plant leaves, which may trigger the inactivation of photosynthesis and leaf chlorosis. Under drought and salt treatments, the chlorophyll contents of the WT and OE lines were decreased compared to that under the normal condition, but lines A and C showed significantly higher chlorophyll content, which were 1.56 and 1.33 folds and 1.39 and 1.33folds of those of WT under the drought and salt treatments, respectively (Figure 4c).

We measured the dynamic water loss rate (WLR) of the detached leaves during the dehydration progress from 0 h to 6 h. The WLR of the WT and OE lines were almost the same before 1 h. From 2 h to 6 h, the average WLR of line A and line C were 13.82–34.89%, which were significantly lower than the 24.30–59.30% of WT (Figure 4d). Malondialdehyde (MDA) is an important indicator of membrane lipid peroxidation and can lead to severe cell membrane damage. Both drought and salt stresses induced MDA accumulation in the WT and OE lines. Under drought treatment, lines A and C accumulated less (86% and 74% of that of WT) MDA than WT. Under salt stress, the MDA content of WT was significantly higher than the line C (1.16 folds), while it was at a similar level with line A (1.06 folds) (Figure 4e). These results indicated that the WT plants suffered more serious membrane damage than did the OE lines (Figure 4e).

Osmotic adjustment mediated by generating osmolytes is an important feature allowing plants to adapt in osmotic stress conditions [36]. We compared the contents of three osmolytes, proline, soluble protein, and soluble sugar, between the WT and OE lines. The results showed that under the drought and salt treatments, lines A and C accumulated more proline (1.20 and 1.59 folds under drought and 1.31 and 1.36 folds under salt), soluble protein (1.36 and 1.25 folds under drought and 1.27 and 1.23 folds under salt) and soluble sugar (1.45 and 1.44 folds under drought and 1.81 and 1.27 folds under salt) than did the WT (Figure 4f–h). Additionally, line A showed a lower proline content but higher soluble protein and soluble sugar content compared to line C.

### 2.5. Effect of MfWRKY40 Overexpression on Antioxidant Metabolism

**Figure 5:**
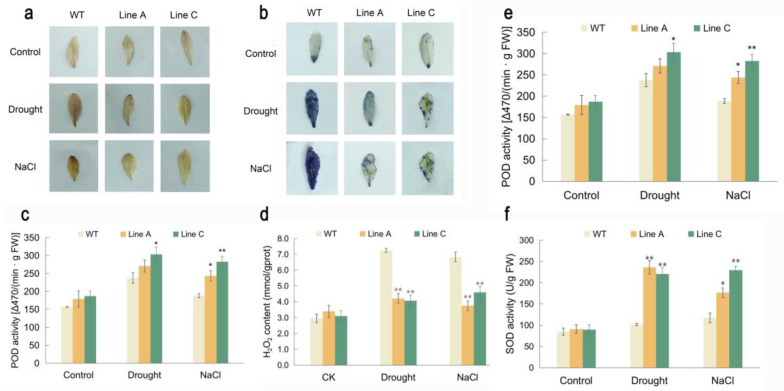
Analysis of ROS accumulation and activities of key antioxidant enzymes under drought and salt treatments. (**a**,**b**) showed the analysis of H_2_O_2_ and O_2_^−^ accumulation by using histochemical staining with DAB and NBT, respectively. (**c**,**d**) showed the content of hydrogen peroxide (H_2_O_2_) and superoxide anion content (O_2_^−^), respectively. (**e**,**f**) indicated activities of peroxidase (POD) and superoxide dismutase (SOD) in the leaves of transgenic and WT plants, respectively. Data are presented as mean and SD values of three independent experiments. Asterisks indicated significant difference (* *p* < 0.05, ** *p* < 0.01, by independent sample *t*-test) compared to WT.

Drought and salt stresses usually cause excessive accumulation of ROS, which will lead to oxidative stress on plant cells. To evaluate the ROS levels under drought and salt treatments, we used 3,3′-diaminobenzidine (DAB) and nitroblue tetrazole (NBT) for histochemical staining. The results showed that under the normal condition, both the WT and OE lines were in similar staining degrees, which were stained in very light brown or only very few parts of the leaves could be stained in blue. These results indicated that the WT and OE lines accumulated similar low contents of H_2_O_2_ and O_2_^−^ under the normal conditions. However, after being treated by drought and salt stresses, the whole leaves of the WT were stained in dark brown or blue by DAB and NBT, respectively, while lines A and C were stained in a visible lighter brown by DAB, and significantly less leaf parts were stained in blue by NBT, respectively (Figure 5a,b). Additionally, we quantified the contents of H_2_O_2_ and O_2_^−^ and found that line A and line C exhibited similar contents of H_2_O_2_ and O_2_^−^ with WT under the normal condition, while significantly lower contents were exhibited under the drought and salt stresses (Figure 5c, d), which were 58% and 56%, 54.94% and 67.35%, 70.74% and 78.11%, and 67.21% and 72.31% of WT, respectively (Figure 5c,d). These results were consistent with those detected by histochemical staining, suggesting that the OE lines overexpressing *MfWRKY40* suffered less oxidative stress.

The peroxidase (POD) and superoxide dismutase (SOD) were the key enzymes involved in ROS scavenging. We determined the activities of the two enzymes and found that under the drought and salt treatments, the activities of POD and SOD of the WT and transgenic plants were both significantly increased. Under drought, the POD and SOD activities of line A and line C were 1.14 and 1.28 folds and 2.33 and 2.18 folds of those of the WT (Figure 5e,f), whereas they were 1.29 and 1.50 folds and 1.51 and 1.96 folds under salt treatment, respectively. These results showed that the lower ROS accumulation in the OE lines was due to, at least partly, the promoted ROS-scavenging system.

### 2.6. MfWRKY40 Promoted Stomatal Closure

**Figure 6:**
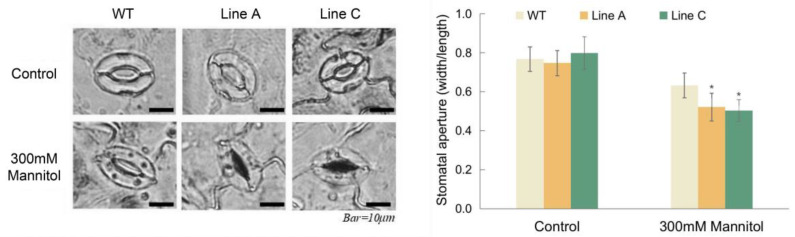
Measurements of stomatal aperture. (**a**) Microscopy observation of the stomatal aperture of OE and WT plants treated by 300 mM mannitol. (**b**) Measurement of the stomatal aperture with or without mannitol treatment. Data are presented as mean and SD values of three independent experiments. Asterisks indicated significant difference (* *p* < 0.05, by independent sample *t*-test) comparing to WT.

We evaluated the stomatal closures of leaves under 300 mM mannitol treatment. Under normal conditions, most of the stomata in the WT and OE lines were open (Figure 6a), and there were no significant differences in the stomatal apertures (width/length ratio) between the OE and WT plants. Mannitol treatment reduced the stomatal apertures of the two OE lines to 0.5 and 0.52, which were significantly lower than the 0.63 of the WT (Figure 6b). These results suggest that MfWRKY40 promotes mannitol-induced stomatal closure.

## 3. Discussion

Up to now, many WRKY TFs have been isolated from different plant species [24,25,37], and are involved in abiotic stress responses. In this study, *MfWRKY40*, which was upregulated at the early dehydration stage [35], was isolated from *M. flabellifolia*. It contains a highly conserved WRKY motif and has the highest homology with a WRKY protein of *Bruguiera gymnorhiza*. The nuclear localization of MfWRKY40 suggests that MfWRKY40 may function as a transcription activator or repressor. It is worth mentioning that there is no zinc finger structure in the WRKY domain of MfWRKY40, which is usually considered as a functional structure binding to the W-box in the promoter region of the target genes with the DNA sequence of (C/T)TGAC(C/T) [38]. We also search the transcriptome dataset of *M. flabellifolia* and failed to find a more intact transcript related to *MfWRKY40* (comp48667_c1__seq16). Therefore, it is unlikely to be derived from alternative splicing.

Previous studies reported that there may be a fourth class of WRKY proteins in which the members contain incomplete WRKY domains. For example, five WRKY proteins found in rice do not contain zinc finger motifs. Whether the five WRKY proteins have regulatory functions have not been studied [39]. The complex and different roles of WRKY40 in response to abiotic stress were also reported [20,22,25,26,27]. In the present study, our results showed that the MfWRKY40 without the zinc finger structure may function as a regulator for drought and salinity tolerance. However, whether this role is related to absence of the zinc finger is unclear.

According to our results, *MfWRKY40* OE plants showed better growth under both drought and salt treatments, indicating that MfWRKY40 plays a positive role in drought and salt responses in *Arabidopsis*. The plant organs, such as the leaves and roots, orchestrate defense mechanisms (internal or external) in response to abiotic stress [40,41,42]. Roots are the first organs exposed to drought and salt stresses because these stresses are inseparable from the soil medium [43,44,45,46,47]. Our results showed that the introduction of *MfWRKY40* into *Arabidopsis* enhanced primary root length under both stress treatments, which may benefit the plant with better water uptaking and transportation under adverse conditions. Additionally, the lower transpiration rate, thick cuticle, and small stomatal aperture enhance the water retention ability and drought tolerance in plants [48]. In our study, the OE lines had smaller stomatal aperture and significantly lower water loss rates. These combined results suggested that MfWRKY40 also enhanced water uptake and retention capacity under stress.

Osmotic adjustment is well known to have an important role in plant adaptation to dehydration via the maintenance of turgor pressure, relative water content, stomatal conductance, and specific cellular functions [49]. The accumulation of compatible solutes in plants is thought to benefit stressed cells, either by acting as cytoplasmic osmolytes to facilitate water uptake and retention or by protecting macromolecules and their structures from stress-induced damage [49]. The common osmotic protective agents in plants include proline, soluble sugar, and soluble protein, etc. All are essential substances for the primary metabolism of plants. Several studies showed that these substances are helpful in plant tolerance when exposed to drought and salinity stresses. Among them, proline affects the genes responsible for stress tolerance and reduces the harmful effects caused by drought stress. It also restores stress injuries by stabilizing the antioxidants system though osmotic adjustments and by decreasing ROS effects [50]. For instance, proline is interconnected with the signaling pathways of the total soluble sugars and the quenching of ROS and thus, promotes drought tolerance in Arabidopsis [51]. Our research shows that under drought or salt stress, the *MfWRKY40* OE lines had higher contents of proline, soluble protein, and soluble sugar than the WT, suggesting that MfWRKY40 may contribute to regulating the accumulation of osmotic substances.

In addition, stresses can increase ROS production and lead to oxidative stress [52]. However, plant cells are well equipped with antioxidants and scavenging enzymes to maintain their levels. By histochemical staining and ROS quantification, we found significantly lower ROS accumulation in OE lines, and the promoted SOD and POD activities were also detected. These results apparently indicated that MfWRKY40 enhanced the ROS-scavenging system. This is similar to the report of *AtWRKY40* overexpression [20].

In conclusion, our results in this study showed that overexpression of *MfWRKY40* of *M. flabellifolia* can enhance the drought and salt tolerance of *Arabidopsis* by directly or indirectly promoting primary root elongation and enhancing water uptake and retention capacity, osmotic adjustment, and antioxidation system. Therefore, it may be involved in positive responses to abiotic stress. However, as MfWRKY40 protein lacks a complete typical WRKY domain, its regulatory mechanisms in abiotic stress responses need to be further investigated.

## 4. Materials and Methods

### 4.1. Plant Materials and Growth Conditions

*M. flabellifolia* used for gene cloning was provided by the Department of Plant Science, University of California, Davis. *Arabidopsis* ecotype *Columbia* (WT) is conserved by our lab. For stress treatment at the seedling stage, the seeds of *Arabidopsis* were sterilized with 50% bleach solution for 5 min and washed with sterile water three to five times, 1 min each time. Then the seeds were evenly planted on square petri dishes (10 × 10 cm) (15 seeds per dish) containing solid 1/2 strength Murashige and Skoog (MS) medium. After 2 days of vernalization in the dark and at 4℃, the medium was placed in a incubator under conditions of 60% relative humidity and long day (16 h light/8 h dark) treatment at 24 °C/22 °C with light intensity of approximately 100 µM photons m^–2^ s^–1^. For stress treatment at the adult stage, WT and transgenic seeds germinated on 1/2 MS medium were planted in pots (8.5 cm deep) with square mouths (10 × 10 cm), containing the same amounts of the substrate (peat soil: vermiculite = 1:1), were grown for four weeks under the same conditions, as mentioned for seedling treatment.

### 4.2. Cloning and Bioinformatic Analysis of MfWRKY40

The total RNA of *M. flabellifolia* was extracted with Plant RNA Kit (Omega). Subsequently, RNA from *M. flabellifolia* was reversely transcribed using Uscript II All in One First-strand cDNA Synthesis Super Mix (InnovGene, Chengdu, China) to obtain the first strand cDNA.

According to the sequence of *MfWRKY40* (uniGene ID: comp48667_c1__seq16) [35], a pair of gene specific primers were designed by using Primer Premier 6 software to isolate the complete coding sequence from cDNA of *M. flabellifolia.* The primer sequences were as follows: forward primer: 5′-TCCCCCGGGATGTCCGAGTCCCTGAACTT-3′ (*Sma* I cut site is underlined) and reverse primer: 5′-GACTAGTTCAGGTGACCTTCTGACCAT-3′ (*Spe* I cut site is underlined). To facilitate the subsequent vector construction, the cleavage sites of two restriction endonucleases, *Sma* I and *Spe* I, were added on the 5′ end of the primers. The primers were synthesized by Qingke Biotechnology (Chengdu, China). Using the cDNA of *M. flabellifolia* as the template, the target gene was amplified by PCR employing Phantamax Super-Fidelity DNA Polymerase (Vazyme Biotech Co., Nanjing, China). The purified PCR product with expected size was cloned into a pEasy-T1 simple vector (Transgenic Biotechnology, Beijing, China) and transformed into *E. coli* strain, DH5 α. The positive clones identified by PCR were sequenced by Qingke Biotechnology Co. (Beijing, China).

The ORF Finder (https://www.ncbi.nlm.nih.gov/orffinder/, accessed on 15 July 2022) was used to find the open reading frame (ORF) of the obtained nucleotide sequence. NCBI blastp search (https://blast.ncbi.nlm.Nih.gov/Blast.cgi, accessed on 15 July 2022) was used to find proteins with high degrees of homology with MfWRKY40. The multiple sequence alignment of these homologous proteins was performed using DNAMAN 9 software. The phylogenetic tree was constructed based on the neighbor joining method using MEGA 7.0 software.

### 4.3. Subcellular Localization of MfWRKY40

A pair of primers was designed to amplify *MfWRKY40* without stop codon and was supplemented with recognition sites of restriction endonucleases *Hind* III and *BamH* I and sequences homologous to the terminals of the pHB-YFP vector containing gene encoding yellow fluorescent protein. The primer sequences were as follows: forward primer: 5′-*ACCAGTCTCTCTCTC*AAGCTTATGTCCGAGTCCCTGAACTT-3′ (*Hind* III site is underlined, homologous arm sequence is italic); reverse primer: 5′-*GCTCACCATACTAGT*GGATCCGGTGACCTTCTGACCAT-3′. (*BamH* I site is underlined; homologous arm sequence is italic). The amplified fragment was cloned into the vector by the homologous recombination method using REIII One-step Cloning Mix (Innovagene biotechnology Ltd., Changsha, China). The resulting construct *35S::MfWRKY40-YFP* and control construct (*35S::YFP*) were respectively transformed into *Agrobacterium tumefaciens* GV3101 by the freeze–thaw method and injected into four-week-old tobacco (*Nicotiana benthamiana*) cells. After 16 h dark treatment and two days of normal cultivation, the leaves of the transformed tobacco were cut and placed under a laser confocal scanning microscope (Nikon, Tokyo, Japan) to observe the expression of YFP in tobacco cells.

### 4.4. Overexpression Vector Construction and Screening of Transgenic Lines

To generate the *35S::MfWRKY40* lines, the coding sequence of *MfWRKY40* was ligated into a binary vector, *pGSA1403*, and transferred into *A. tumefaciens* LBA4404. Subsequently, the transgenic *Arabidopsis* was produced by the floral-dip transformation method [53]. The T_0_ seeds were collected and screened on 1/2 MS medium supplied with kanamycin (50 μg/mL). The continuous selfing and screening with kanamycin and PCR detection were performed. The resulting T_3_ homozygous positive lines were randomly selected for further analysis.

### 4.5. Drought and Salinity Treatments

For drought and salt treatments at the seedling, sterilized seeds of WT and two T_3_ transgenic lines were germinated and vertically grown in square petri dishes containing 1/2 MS medium containing varying concentrations of mannitol (0 mM, 200 mM, 250 mM and 300 mM) and NaCl (0 mM, 50 mM, 75 mM and 100 mM). The growing conditions were mentioned above. After nine days, the primary root lengths of each line were measured. A total of 15 seedlings from each line were measured, and three biological repeated experiments were conducted.

The four-week-old plants of the WT and transgenic lines were used to explore the tolerance of mature plants to drought and salt stresses. The plants grown in pots were saturated with water before treatment. For drought stress, the watering was held for 19 days and then rewatered for three days. For salt treatment, plants were irrigated by 300 mM NaCl solution twice at three-day intervals. The whole treatment was continued for 15 days. The plants were observed and photographed regularly. Each treatment was performed in three biological repeats.

### 4.6. Determination of Water Loss Rate

The WT and transgenic plants were conventionally cultivated for four weeks. The 0.5 g rosette leaves were cut from similar positions of the WT plants and two T_3_ transgenic lines and weighed immediately. Then the leaves were placed on filter paper at room temperature (~24 ℃, 60% relative air humidity) and weighed at set time points (0, 1, 2, 3, 4, 5, and 6 h). The water loss rate representing percentage of water loss at each time point were obtained according to the fresh weights of the leaves before dehydration. The experiment was repeated three times.

### 4.7. Physiological Index Determination

Chlorophyll was directly extracted with 95% ethanol and quantified as previously reported [54]. The acidic ninhydrin colorimetric method was used for the determination of proline content [55]. Soluble sugar and soluble protein were quantitatively determined with Plant Soluble Sugar Content Detection Kit (Nanjing Jiancheng, Nanjing, China) and Total Protein Quantitative Determination Kit (Nanjing Jiancheng, Nanjing, China), respectively. The histochemical staining using 3,3′-diaminobenzidine (DAB) and nitroblue tetrazole (NBT) was used for visualization of the accumulation of hydrogen peroxide (H_2_O_2_) and superoxide anion radical (O_2_^−^) in the leaves, respectively [56]. A hydrogen peroxide assay kit and superoxide anion assay kit (Nanjing Jiancheng, Nanjing, China) were used to measure the contents of H_2_O_2_ and O_2_^−^, respectively. The SOD activity was determined by the diazo-blue tetrazole photo-reduction method [57]. The POD activity was determined by the guaiacol determination method [58]. The malondialdehyde (MDA) content of each strain was determined with the thiobarbituric acid method [59]. Three replicates were executed for all these experiments.

### 4.8. Stomatal Aperture Analysis under Drought Stress

The rosette leaves of four-week-old plants of the WT and transgenic lines were placed in the MES–KCl solution (50 mM KCl, 0.1 mM CaCl_2_ and 10 mM MES, pH = 6.15) and exposed to light for 2.5 h. The treated leaves were then transferred to MES–KCl solutions containing 0 and 300 mM mannitol for 2 h of exposure to light, respectively. The stomata on the lower epidermis of the leaves were immediately observed and photographed by an optical microscopy (DP80, Olympus, Japan). The width–length ratios of at least 50 stomatal cells was measured. All experiments were repeated three times.

### 4.9. Statistical Analysis

Data were analyzed by Student’s *t*-test using SPSS v. 23.0 and shown as the mean ± standard deviation (SD) of three replicates. The significance of difference was expressed as * (*p* < 0.05) or ** (*p* < 0.01).

## Data Availability

Not applicable.
